# Expression and characterization of Est2: a novel cold-adapted esterase from Antarctic bacterium defining the new esterase family XXII

**DOI:** 10.3389/fmicb.2025.1662394

**Published:** 2025-09-05

**Authors:** Yuanfang He, Zeyuan Sun, Xiying Zhang, Shu Xing, Hailun He, John Kevin Bielicki, Mingyang Zhou

**Affiliations:** ^1^School of Chemistry and Chemical Engineering, Qilu University of Technology (Shandong Academy of Sciences), Jinan, China; ^2^State Key Laboratory of Microbial Technology, Shandong University, Qingdao, China; ^3^School of Life Sciences, State Key Laboratory of Medical Genetics, Central South University, Changsha, China

**Keywords:** Antarctic, bacteria, esterase, cold-adapted, new esterase family XXII

## Abstract

Extremozymes from Antarctic microbiota represent a potential source of unique biocatalysts. In this study, a novel esterase gene *est2* was identified from the Antarctic bacterium *Pseudomonas* sp. A6-5. Phylogenetic and sequence analyses classified it as the founding member of a new esterase family XXII. The catalytic triad of the enzyme consisted of Ser^141^, Asp^275^, and His^303^, with the nucleophilic Ser^141^ situated within the characteristic GXSXG motif of α/β-hydrolases. Est2 exhibited remarkable cold-adaptation where 20–85% of the maximum activity was observed at temperatures ranging from 0 to 15°C. Substrate specificity profiling revealed preferential hydrolysis of medium-chain *p*-nitrophenyl esters and triglyceride emulsions. Enzyme activity was sensitive to inhibition by transition metals (1 mM of Mn^2+^, Cu^2+^, Co^2+^, Ni^2+^ or Zn^2+^), but alkali metals were considerably less effective. Representative polar-protic and -aprotic solvents uniformly inhibited Est2 activity. Collectively, these results suggest the structural stability of Est2 is largely governed by hydrophobic interactions and H-bonding, rather than ionic forces. Est2 appears to represent a unique cold-adaptive enzyme that may be suitable for bio-catalyzed environmental remediation.

## 1 Introduction

Esterases (carboxylic ester hydrolases, EC 3.1.1.1) represent a functionally important group of enzymes that catalyze the hydrolysis and synthesis of ester bonds, playing crucial roles in various biological processes (Bornscheuer, 2002). Characterized by their α/β hydrolase fold and conserved catalytic triad (Ser-Asp-His), these enzymes exhibit remarkable region- and stereospecificity without requiring cofactors, making them ideal biocatalysts (Kim et al., 2007; Javed et al., 2018). Their ability to remain stable and active in organic solvents further enhances their industrial value across multiple areas, including detergent formulations for cold-water washing, flavor ester production in food technology, synthesis of chiral intermediates for pharmaceuticals, and polyester biodegradation (Panda and Gowrishankar, 2005; Li et al., 2022; Rafeeq et al., 2022). The broad substrate specificity of esterases, ranging from short-chain fatty acid esters to complex lipids, along with their high enantioselectivity, makes them indispensable tools in both industrial and biomedical fields. Their commercial importance continues to grow as they meet the increasing demand for natural and sustainable bioprocesses.

The classification of bacterial esterases has undergone progressive refinement alongside the accumulation of sequence and structural data. The initial taxonomic framework (Arpigny and Jaeger, 1999) categorized bacterial lipolytic enzymes into eight distinct families (I-VIII) complemented by six true lipase subfamilies (I.1–I.6). This work was based on conserved sequence motifs and characteristic biochemical properties. This classification scheme was subsequently expanded (Kovacic et al., 2018) through comprehensive phylogenetic analysis, extending the taxonomy to 19 principal families (I–XIX) with eight lipase subfamilies (I–VIII). Most recently, the identification of novel enzymes has driven further development of this classification system. These enzymes include the chlorpyrifos-hydrolyzing carboxylesterase EstC (Wang et al., 2020) and the cold-adapted esterase Est33 from Antarctic microbiota (Liu et al., 2022). The latter discoveries led to the formal establishment of two additional families, designated Family XX and Family XXI, completing the current 21 family classification scheme. This evolving taxonomy reflects both the considerable diversity of microbial esterases and the ongoing identification of novel enzymatic variants through modern genomic approaches.

Psychrophilic microorganisms inhabiting extreme environments such as Antarctica have evolved specialized cold-adapted enzymes to maintain metabolic activity under permanently low temperatures, high salinity, and intense UV radiation (Gomes and Steiner, 2004). Other compositional biases observed in psychrophilic proteins include increased asparagine, lysine, methionine and glycine contents, with glycine clustering at the enzyme catalytic site – a feature that increases local mobility and contributes to their cold adaptation. These extremozymes exhibit distinct structural adaptations including reduced proline content, elongated surface loops, and increased number and size of enzyme cavities, which collectively confer enhanced catalytic efficiency at temperatures approaching 0°C (Chiuri et al., 2009; De Maayer et al., 2014; Tribelli and López, 2018; Kamaruddin et al., 2022). Compared to their mesophilic counterparts, these enzymes demonstrate greater molecular flexibility, particularly in their active sites, along with lower activation energy requirements, but at the cost of reduced thermal stability. Such unique properties make cold-adapted enzymes particularly valuable for diverse biotechnological applications including low-temperature industrial processes and environmental bioremediation in polar ecosystems (Ashaolu et al., 2025). Furthermore, their ability to catalyze reactions under mild conditions makes them ideal for the synthesis of thermolabile compounds in pharmaceutical manufacturing, offering sustainable alternatives to conventional high-energy processes (Nandanwar et al., 2020).

In the present study, we identified a novel esterase, Est2, from *Pseudomonas* sp. A6-5 isolated from Antarctic soil (Liu J. et al., 2021). Phylogenetic and structural analyses revealed that Est2 exhibits significant divergence from established esterase families. The enzyme forms a distinct evolutionary clade and shows low sequence identity with members of known families. Based on these molecular characteristics and its Antarctic origin, we propose Est2 as the founding member of a new esterase family, designated Family XXII. Furthermore, we performed structural analyses and compared it with EstC, the thermophilic founding member of Family XX which exhibits high thermal stability, to gain deeper insights into Est2’s cold-adaptation mechanisms. Comparative analyses demonstrated that, compared to EstC (Wang et al., 2020), Est2 contains more exposed hydrophobic residues, longer catalytic loops, reduced proline content but increased amounts of unstable amino acid residues such as asparagine, lysine and methionine, and possesses larger cavities and additional tunnels. This discovery expands the known diversity of bacterial esterases and provides insights into cold-adaptation mechanisms.

## 2 Materials and methods

### 2.1 Bacterial strain and gene source

The *est2* gene, was identified from the genome sequence of *Pseudomonas* sp. A6-5, obtained from Antarctic soil on King George Island (62°11′17.5”S, 58°55′23.4”W). The identity of the *Pseudomonas* strain was verified through 16S rRNA gene sequencing and phylogenetic analysis. The *est2* gene was selected for further study due to its high similarity to esterase genes in the alpha-beta hydrolase superfamily and its potential cold-adaptive properties.

### 2.2 Sequence and phylogenetic analysis

The *est2* gene sequence was analyzed using ORF Finder (NCBI)^1^ to identify the open reading frame (ORF). Signal peptides were predicted with SignalP-6.0^2^ (Teufel et al., 2022), and the protein structure was modeled using AlphaFold2 (Jumper et al., 2021). Homologous protein sequences were retrieved from the NCBI database using BLAST for subsequent alignment.^3^ Conserved domains, including the GXSXG motif and catalytic triad, were identified through sequence alignment with Clustal X (Jeanmougin et al., 1998) and visualized using ESPript 3.0.^4^ Reference sequences from GenBank and UniProt were used for phylogenetic analysis. The phylogenetic analysis included 2–3 representative sequences per esterase family, selected from the classification frameworks established in (Wang et al., 2020) and (Kovacic et al., 2018), and the phylogenetic tree was constructed using the maximum likelihood method in IQ-TREE (Trifinopoulos et al., 2016) with 1,000 bootstrap replicates and visualized using iTOL v6^5^ (Letunic and Bork, 2024). The GenBank accession number of the *est2* gene was OR552631.

### 2.3 Gene cloning and vector construction

The *est2* gene was cloned into the pMAL-c2x vector containing an MBP-tag The MBP tag was used because it enhances the solubility of the recombinant protein (~40 kDa) and facilitates subsequent purification. Primers were designed based on the est2 sequence and the vector’s MCS, incorporating *Eco*RI and *Hin*dIII restriction sites: 5′- CAACCTCGGGA TCGAGGGAAGGATG ACCCCTTCTCCCGACCGCT-3′ and 5′- ACGACGGCCAGTGCCAAGCTT TTACTGACCCG AAGCGGGCGAT-3′ (restriction sites underlined). The *est2* gene was amplified by PCR using *Pseudomonas* sp. A6-5 genomic DNA as the template. The target DNA fragment was excised and purified using a Gel Extraction Kit (Omega bio-tek, Guangzhou, China). The pMAL-c2x vector was linearized by double digestion with *Eco*RI and *Hin*dIII restriction enzymes (Thermo Fisher Scientific, Waltham, MA, USA) and the linearized vector was purified.

The purified est2 fragment and linearized vector were ligated using the NovoRec^®^ Plus PCR One-Step Direct Cloning Kit (Novoprotein, Shanghai, China) at 50°C for 15 min. The recombinant plasmid was transformed into *Escherichia coli* BL21 (DE3) for protein expression.

### 2.4 Protein expression and purification

The recombinant *E. coli* BL21 (DE3) strain was inoculated at 1% (*v*/*v*) into 100 mL of LB medium supplemented with ampicillin (100 μg/mL) and cultured at 37°C with agitation at 180 rpm until the OD_600_ reached the range of 0.6–0.8. The culture was chilled on ice, and protein expression was induced by the addition of isopropyl-β-d-thiogalactopyranoside (IPTG) to a final concentration of 0.1 mM, followed by incubation at 17°C with shaking at 120 rpm for 48 h.

After induction, the cells were harvested by centrifugation at 12,000 rpm for 3 min at 4°C. The cell pellet was suspended in CB buffer (20 mM Tris–HCl, 0.2 M NaCl, 1 mM EDTA, pH 7.5) and lysed by sonication. The lysate was centrifuged at 12,000 rpm for 20 min at 4°C, and the supernatant containing the crude enzyme was collected.

The MBP-tagged fusion protein was purified by amylose resin affinity chromatography (New England Biolabs, Beijing, China). The resin was pre-equilibrated with CB buffer, and the crude enzyme extract was filtered through a 0.22 μm membrane before loading onto the column. Non-specifically bound proteins were removed by washing with 10 column volumes of CB buffer, and the target protein was eluted using EB buffer (10 mM maltose, 20 mM Tris–HCl, 0.2 M NaCl, 1 mM EDTA, pH 7.5). Fractions exhibiting high purity, as determined by SDS-PAGE, were pooled and dialyzed overnight at 4°C against 50 mM Tris–HCl (pH 8.0).

To remove the MBP tag, the purified fusion protein was incubated with Factor Xa protease (2% w/w) at 23°C for 6 h in 50 mM Tris–HCl (pH 8.0). The reaction mixture was subsequently subjected to a second round of amylose resin chromatography to separate the cleaved MBP tag and residual protease. The final purified protein was analyzed by SDS-PAGE to confirm purity and integrity. N-termini sequencing was performed by a commercial company (Biotech-Pack, China).

### 2.5 Esterase assay

The protein concentration of the purified, recombinant Est2 enzyme was determined using the Bradford assay with bovine serum albumin as the standard (Kielkopf et al., 2020). Esterase activity was measured spectrophotometrically as the release of *p*-nitrophenol (*p*NP) from the hydrolysis of *p*NP esters (Liu X. et al., 2021). Initial experiments utilized pNP esters of varying acyl chain lengths, including acetate (C2), butyrate (C4), hexanoate (C6), octanoate (C8), decanoate (C10), dodecanoate (C12), and palmitate (C16), prepared in isopropanol. Subsequent experiments utilized the C8 substrate (i.e., standard reaction conditions) to characterize Est2 activity, because it yielded maximal enzymatic activity. The standard reaction mixture contained 20 μL of 10 mM p-nitrophenyl octanoate (C8), 20 μL of enzyme solution (25.2 μg protein), and 0.96 mL of Tris–HCl buffer (50 mM, pH 8.0) to achieve a final volume of 1 mL. The reaction was carried out in a water bath at 30°C for 10 min and terminated by adding 100 μL of 10% SDS. The release of *p*-nitrophenol was quantified by measuring the absorbance at 405 nm. A blank control was prepared by replacing the enzyme solution with an equal volume of buffer. All experiments were performed in triplicate, with the highest activity under optimal conditions was defined as 100% for relative activity calculations.

Temperature and pH optima were determined using the standard assay conditions (C8; 0.2 mM). For temperature studies, reactions were assessed at intervals of 10°C within a range of 0 to 90°C. To evaluate the optimal pH, Britton-Robinson buffer was used to adjust the pH of the reaction system at 30°C, covering a pH range of 4.0 to 11.0 with intervals of 1.0 or 0.5. To investigate the thermal stability of the enzyme, Est2 esterase was incubated at 20°C, 30°C and 40°C for 120 min, and the residual activity was measured every 15 min.

Hydrolysis of triacylglycerol substrates was determined using tributyrin, tricaprylin, and trilaurin as substrates following a titration assay as described (Li et al., 2020). Triacylglycerol emulsions were prepared at a final concentration of 10 mM in 2.5 mM Tris–HCl (pH 7.0), 100 mM NaCl, and 1% (w/v) gum arabic, with pH adjusted to 7.00 using 5 mM NaOH. The reaction mixture consisted of 20 μL triacylglycerol emulsion and 0.5 mL enzyme solution, while a blank control replaced the enzyme with an equal volume of buffer. After incubation at 30°C for 30 min, reactions were terminated by adding 5 mL ethanol. The released fatty acids were quantified by measuring the volume of 5 mM NaOH required to neutralize the reaction mixture. Relative activity was determined by assigning 100% to the substrate eliciting the highest NaOH consumption, with activities toward other substrates normalized proportionally. The data are the mean values of three independent experiments performed in triplicate. Error bars show the standard deviation (SD).

### 2.6 Effect of metal ions and organic solvents on the esterase activity

The effects of metal ions (Li^+^, K^+^, Mg^2+^, Ca^2+^, Mn^2+^, Fe^2+^, Co^2+^, Ni^2+^, Cu^2+^, Zn^2+^, and Ba^2+^) on the activity of Est2 were investigated using the standard reaction mixture containing either 1 mM and 10 mM each of the respective ions.

The stability of Est2 in the presence of organic solvents, including methanol, formaldehyde solution, ethanol, acetonitrile, acetone, isopropyl alcohol, and dimethyl sulfoxide (DMSO), was evaluated at solvent concentrations of 20 and 40% (*v*/*v*). The enzymatic activity was measured under standardized conditions.

### 2.7 Comparison of thermophilic carboxylesterase EstC

The structures of both EstC and Est2 were predicted using AlphaFold2 (Jumper et al., 2021). Hydrophobic surfaces were generated and displayed using the Molecular Lipophilicity Potential tool in ChimeraX (Pettersen et al., 2021). The percentages of proline, asparagine, lysine, and methionine were determined using the ExPASy database (Gasteiger et al., 2005). Polar and non-polar accessible surface areas were calculated with the online software VADAR (Willard et al., 2003). Pocket volume measurements were performed by analyzing the predicted structures with CASTpFold (Ye et al., 2024). Amino acid residues and catalytic loops were visualized using PyMOL^6^.

## 3 Results

### 3.1 Sequence analysis and phylogenetic analysis

**Figure 1:**
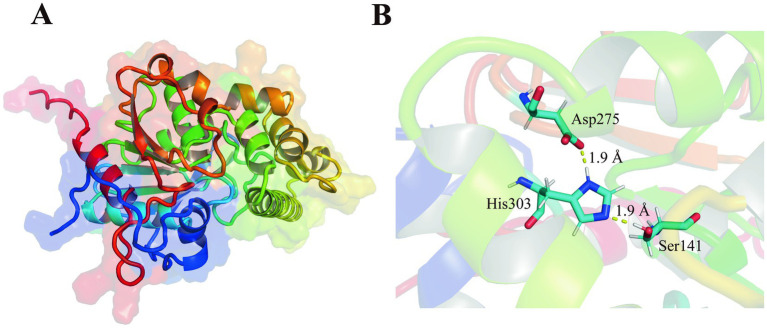
Structures of new family esterase. **(A)** Structural modeling of Est2 was performed using AlphaFold2, and the optimal model was selected for visualization with PyMOL. **(B)** Structural analysis of Est2 revealed a canonical catalytic triad comprising Ser^141^ (nucleophile), Asp^275^ (charge relay), and His^303^ (proton carrier) within its active site. The distances between catalytic Ser and His, His and Asp of Est2 were 1.90 Å, 1.90 Å, respectively.

The est2 gene was comprised of an open reading frame (ORF) of 1,017 base pairs. The gene encoded a polypeptide of 338 amino acids with no signal peptide. The later suggests a probable intracellular localization. The predicted three-dimensional structure of Est2 exhibited a canonical α/β hydrolase fold, aligning with its functional classification as an esterase (Figure 1).

**Figure 2:**
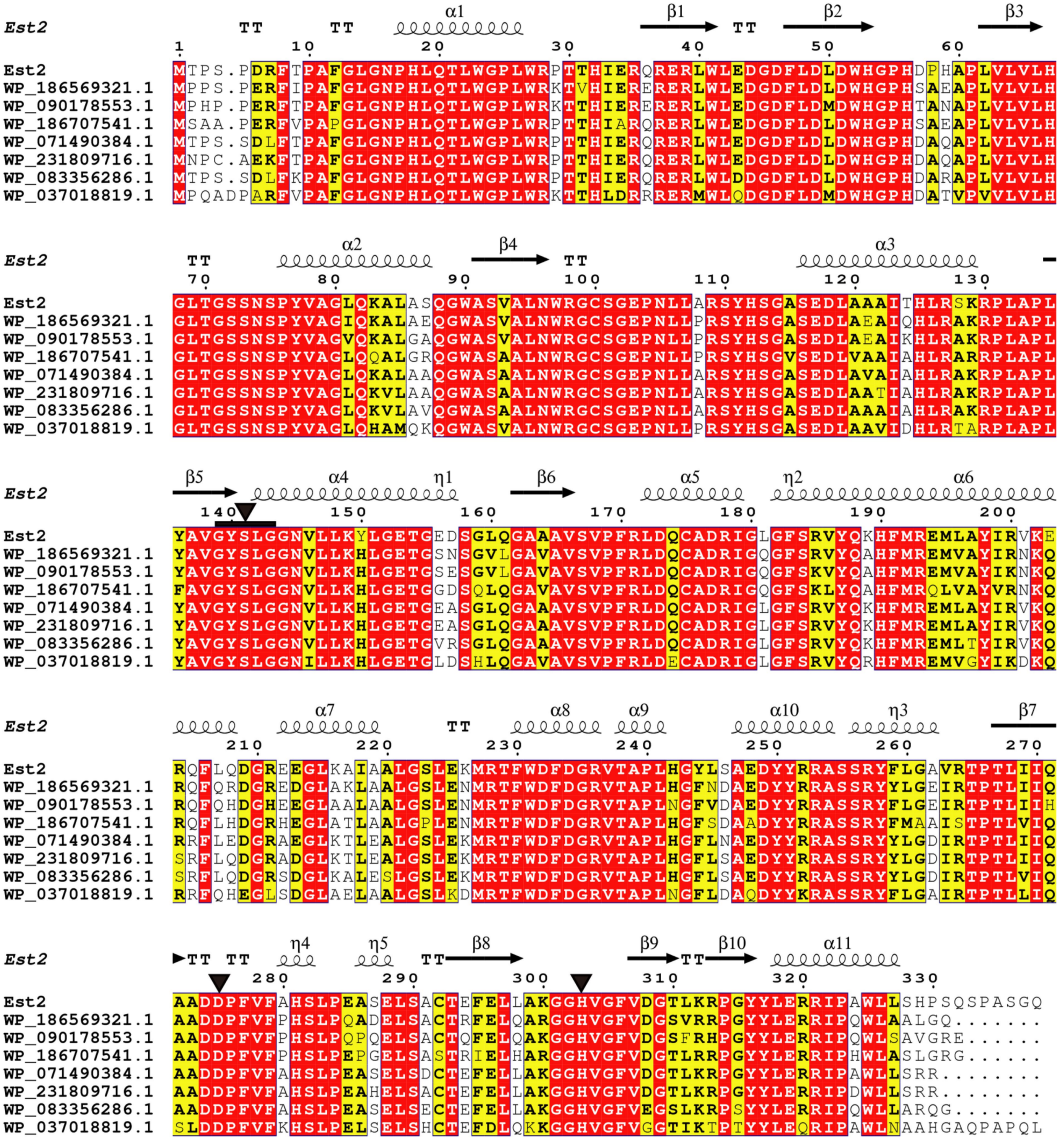
Sequence alignment of Est2 with homologous proteins. Identical residues appear as white text on red background, while similar residues are shown in bold black within yellow boxes. The secondary structure elements of Est2 are annotated above the sequences: α-helices (spring symbols), β-strands (arrows), turns (TT), and 3_10_ helices (*η*). Catalytic triad residues (Ser, Asp., His) are marked with black triangles, and conserved G-X-S-X-G pentapeptides are indicated by black squares.

Protein BLAST analysis identified the top 100 amino acid sequences with the highest similarity as uncharacterized α/β hydrolases annotated from *Pseudomonas* genomes. Among these, seven sequences displaying varying degrees of identity to Est2 were selected for detailed multiple sequence alignment. This alignment revealed conserved structural motifs characteristic of esterases, including the GXSXG motif and the catalytic triad formed by Ser^141^, Asp^275^, and His^303^ (Figure 2). These critical catalytic residues are highly conserved across esterase families.

**Figure 3:**
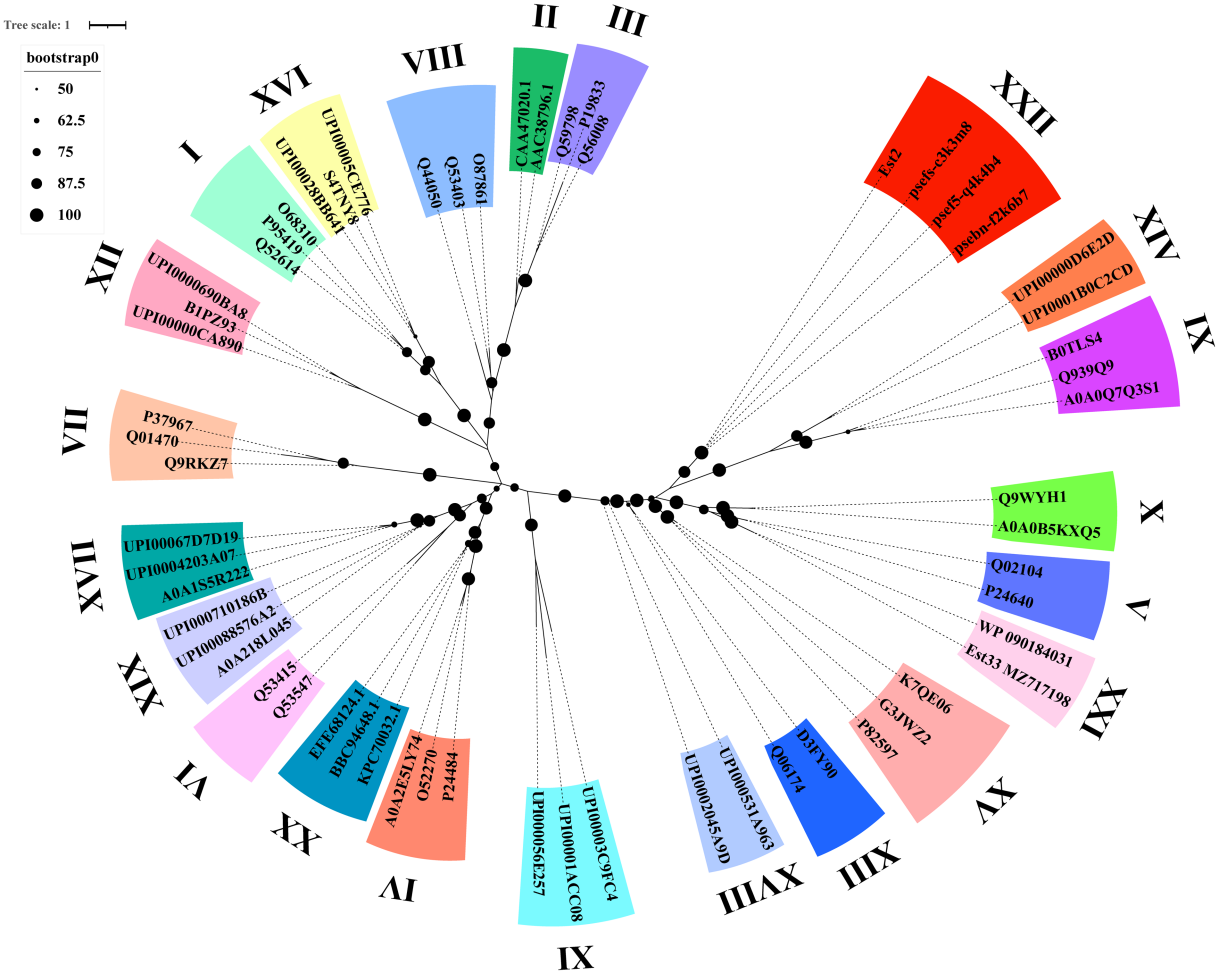
Phylogenetic analysis of Est2, its homologs, and other esterase families. The Maximum Likelihood tree was generated using IQ-TREE web-server with automatic model selection and visualized in iTOL v6. Bootstrap values (>50%) from 1,000 replicates are displayed. Protein sequence sources are provided in Supplementary Table S1.

Phylogenetic analysis demonstrated that Est2 clustered to a distinct clade, separate from previously characterized esterase families, establishing it as the founding member of a novel esterase family, designated as family XXII (Figure 3). This classification is supported by high bootstrap values, confirming the evolutionary divergence of Est2 from other esterase families.

### 3.2 Expression and purification of Est2

**Figure 4:**
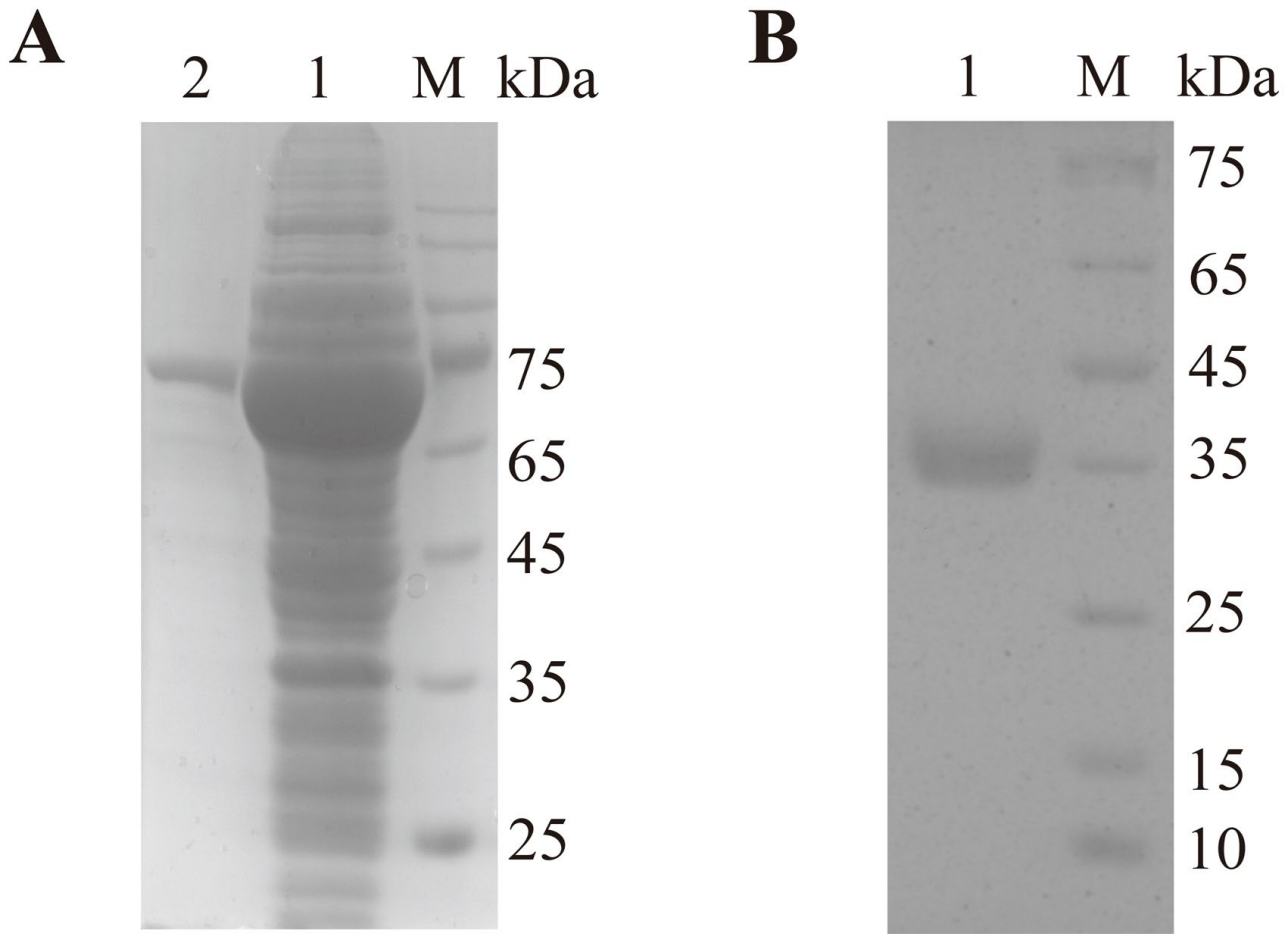
SDS-PAGE analysis of Est2. Lane M, protein molecular mass marker. **(A)** After purification through maltose affinity chromatography, the electrophoresis image of the esterase Est2 with an MBP tag. **(B)** After Factor Xa protease cleavage of the MBP tag and further purification by affinity chromatography, the electrophoresis image of the pure enzyme Est2.

The *est2* gene was successfully expressed in *Escherichia coli* BL21 (DE3) as an MBP-tagged fusion protein, which improved solubility and facilitated protein purification. Following induction with IPTG, the recombinant protein was purified using amylose resin affinity chromatography, yielding a protein of high purity as confirmed by SDS-PAGE (Figure 4A). The MBP tag was subsequently removed by Factor Xa protease treatment, and the final purified Est2 protein exhibited a molecular weight of approximately 37 kDa, consistent with theoretical predictions (Figure 4B). SDS-PAGE analysis demonstrated the successful purification of Est2, with no significant contamination from host proteins or residual MBP tag.

### 3.3 Biochemical characterization of Est2

**Figure 5:**
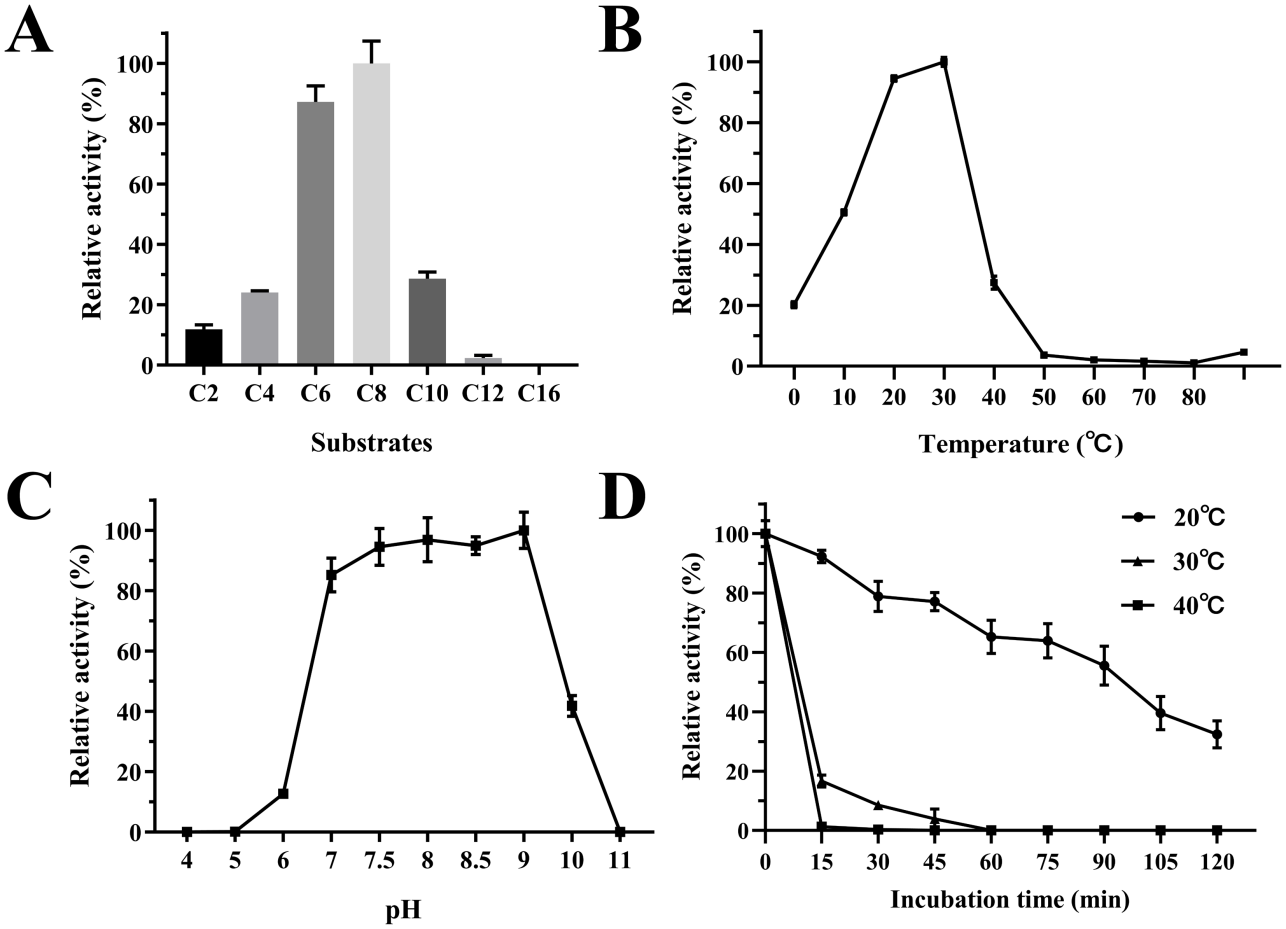
Biochemical characterization of Est2. **(A)** Determination of Est2 substrate specificity. The relative activity was calculated assuming the highest activity observed with *p*-nitrophenyl octanoate (C8; 0.2 mM) as 100%. **(B)** Optimum temperature for Est2 activity with *p*NPC8 (0.2 mM). **(C)** Determination of the optimum pH for Est2 activity with *p*NPC8 (0.2 mM). **(D)** Enzyme thermostability for Est2 activity with *p*NPC8 (0.2 mM).

Est2 exhibited the highest catalytic activity toward *p*-nitrophenyl octanoate (C8), with significantly lower activity observed for longer-chain substrates such as *p*-nitrophenyl dodecanoate (C12) and *p*-nitrophenyl palmitate (C16) (Figure 5A). This substrate preference confirms Est2 as an esterase rather than a lipase, as it preferentially hydrolyzes medium-chain esters.

The optimal temperature for Est2 activity was determined to be 30°C, with significant activity observed between 10°C and 30°C. Remarkably, Est2 retained approximately 20% of its activity at 0°C, highlighting its cold-adapted nature (Figure 5B). The enzyme exhibited maximal activity over a pH range of 7–9, while retaining measurable activity across a broad pH range pH 6–10 (Figure 5C).

Treatment of Est2 at various temperatures for relatively prolonged periods also revealed its cold-adaptive nature. Approximately 40% of Est2 activity was retained following 2-h incubation at 20°C. In contrast, enzymatic activity decreased rapidly when incubated at 30°C and 40°C, with significant inactivation observed within just 15 min (Figure 5D). These results indicated that Est2 is more stable at lower temperatures.

**Figure 6:**
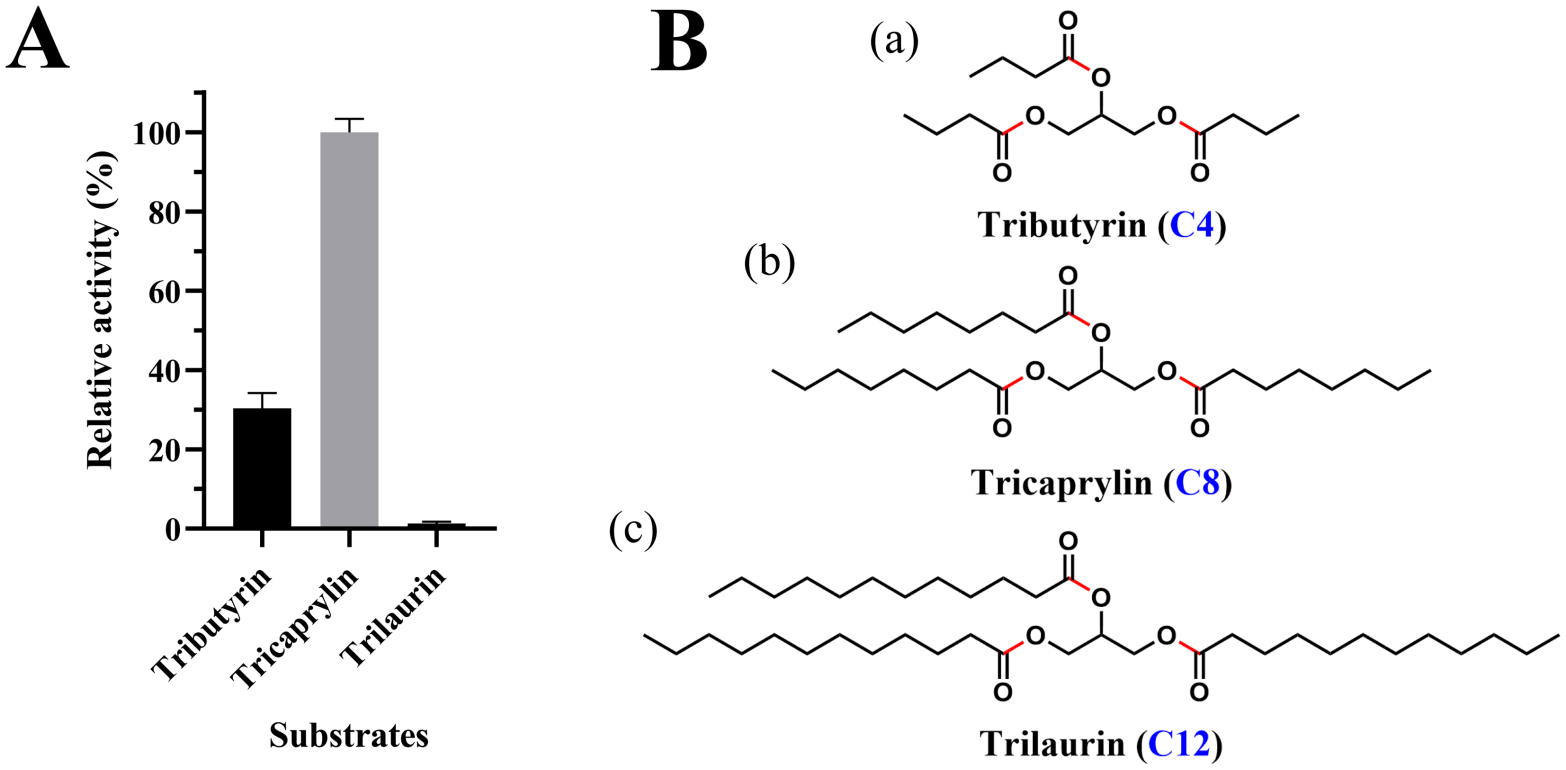
Triacylglycerol substrate specificity and structural representation of Est2. **(A)** Relative lipase activity of Est2 toward tributyrin, tricaprylin, and trilaurin (10 mM). Relative activity was calculated by defining the substrate with maximal NaOH consumption (tricaprylin; 10 mM) as 100%, with activities toward other substrates normalized proportionally to the NaOH volume required for tricaprylin hydrolysis. In all panels, the data are the mean values of three independent experiments performed in triplicate. Error bars, standard deviation (SD). **(B)** Molecular structures of substrates. **(a)** Tributyrin, **(b)** tricaprylin, **(c)** trilaurin. Key structural features are indicated: The glycerol backbone (black) is esterified with three fatty acid chains. Carbon chain lengths of fatty acid constituents are labeled with blue numerals. Red highlights indicate the ester bonds.

Est2 exhibited detectable hydrolytic activity toward triacylglycerol substrates, with maximal activity observed for tricaprylin, followed by tributyrin, while no activity was detected for trilaurin (Figure 6A). The chemical structures of these substrates (Figure 6B) highlight the chain-length dependence of Est2’s activity, demonstrating its preference for medium-chain triglycerides (C4-C8) over the long-chain trilaurin (C12). This substrate selectivity profile aligns with the canonical functional definition of esterases, which typically hydrolyze acyl chains shorter than C10, as further supported by the complete absence of activity toward the C12 substrate in Figure 6A. The combined evidence from catalytic activity and structural analysis unequivocally supports classifying Est2 as a cold-adapted esterase rather than a lipase.

### 3.4 Effects of metal ions and organic solvents

**Figure 7:**
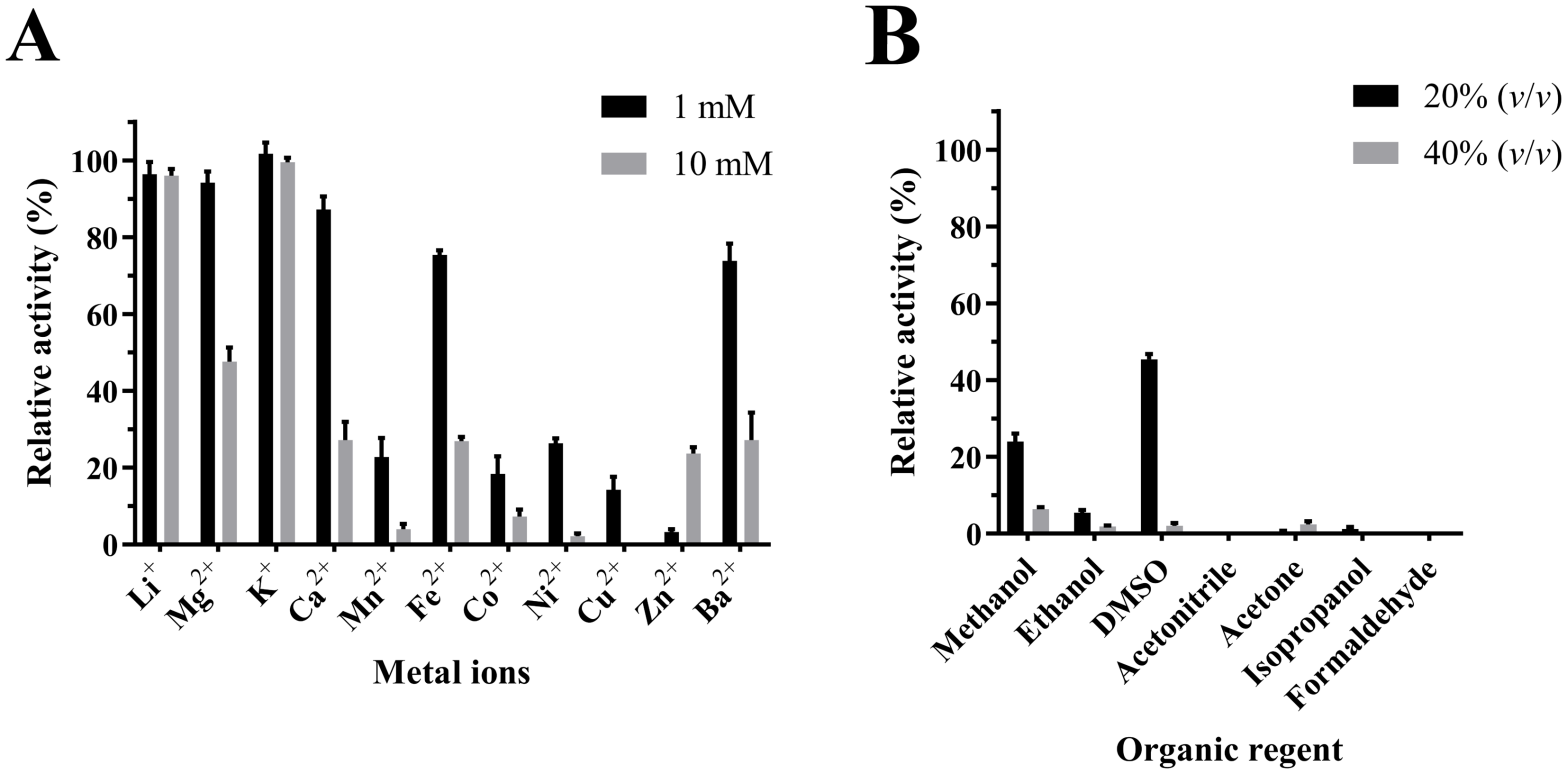
Effects of Metal Ions and Organic Solvents on the activity of Est2. **(A)** Effect of metal ions on Est2 activity. Relative activity of Est2 in the presence of several cationic metal ions (1 mM and 10 mM). **(B)** Est2 activity in the presence of Organic solvents (20 and 40%). For all panels, the activity of Est2 was determined with *p*-nitrophenyl octanoate (C8; 0.2 mM). The data are the mean values of three independent experiments performed in triplicate. Error bars, standard deviation (SD).

The influence of metal ions on the enzymatic activity of Est2 was investigated (Figure 7A) by comparing potential inhibitory effects of alkali metals versus transitions metals. Alkali metals, such as K^+^ and Li^+^, had little effect on Est2 activity at concentrations of 1 and 10 mM; while, the alkali earth metals, such as Mg^2+^, Ca^2+^, and Ba^2+^, were only weakly effective. These results suggest ionic interactions are not likely a major driving force governing enzyme stability. In contrast, significant inhibition was observed with transition metals (Mn^2+^, Co^2+^, Ni^2+^, Cu^2+^, and Zn^2+^). Notably, pronounced inhibitory effects were seen using 1 mM concentrations of Cu^2+^, Zn^2+^, and Co^2+^, suggesting these metal ions may act as potential inhibitors of Est2.

The ability of Est2 to function in the presence of various polar solvents was evaluated (Figure 7B). The enzyme demonstrated the highest tolerance to aprotic solvent DMSO, retaining approximately 45% of its activity at 20% (*v*/*v*) concentration. Overall, however, Est2 appeared sensitive to inhibition by other polar solvents; with only modest activity remaining at 20 and 40% (v/v) methanol.

### 3.5 Comparative analysis with thermophilic enzyme EstC

**Figure 8:**
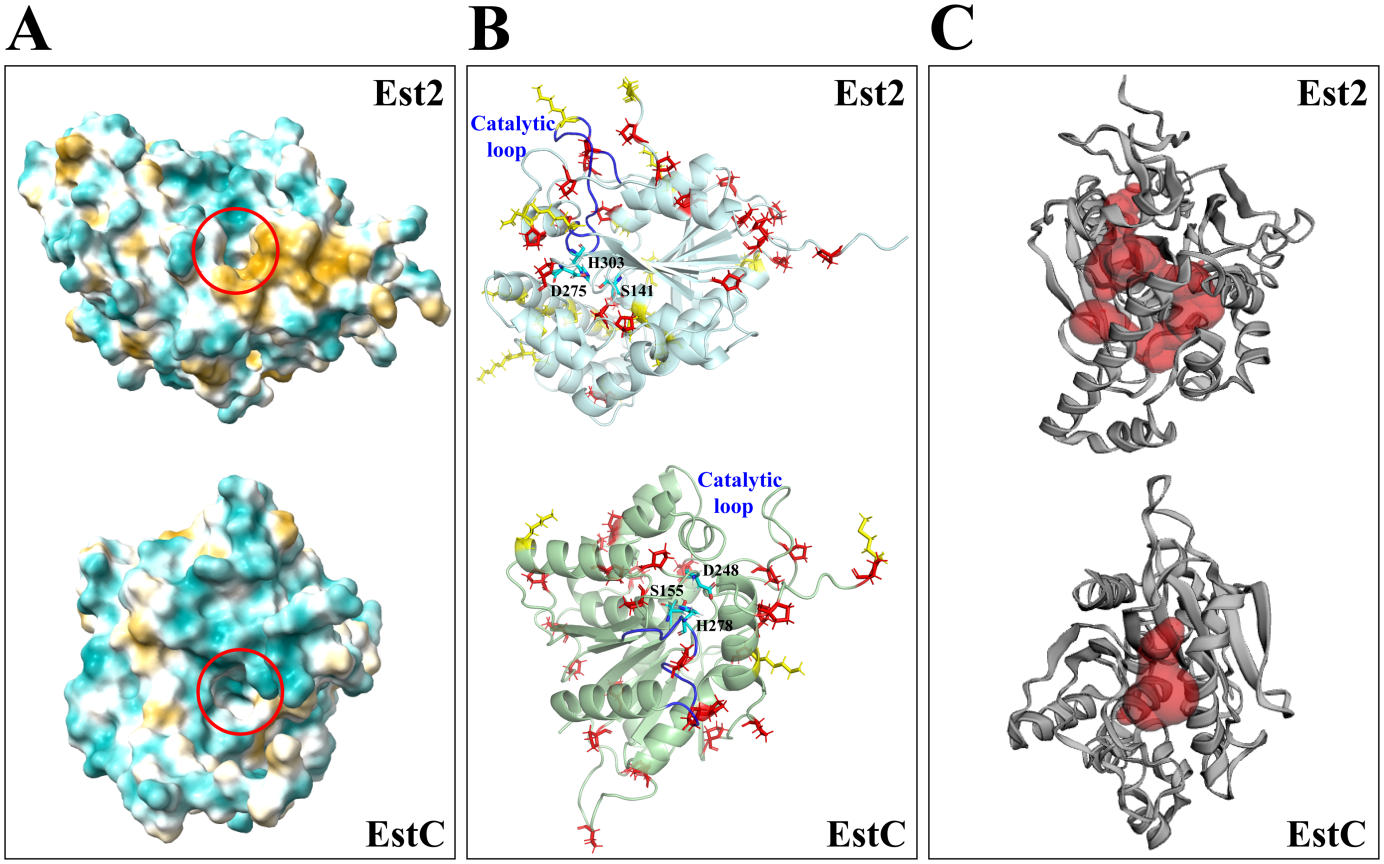
Structural comparison of cold-adapted Est2 and thermophilic EstC. **(A)** The hydrophobic surfaces generated by ChimeraX, with a hydrophobicity gradient ranging from hydrophilic (deep cyan) to hydrophobic (deep yellow). Catalytic cavities are indicated by red circles. **(B)** Cartoon representations of Est2 and EstC. Stabilizing proline residues (Pro) are shown as red sticks, destabilizing residues (Asn, Lys, and Met) as yellow sticks, and catalytic triad residues in cyan stick representation. Catalytic loops in Est2 and EstC are colored blue. **(C)** Binding pockets and cavities of Est2 and EstC identified by CASTp 3.0, with pockets highlighted in red.

The comparative structural analysis revealed distinct cold-adaptation features in Est2 when compared to the thermophilic EstC. Est2 displayed an altered amino acid composition characterized by increased proportions of destabilizing residues such as asparagine, lysine and methionine, along with reduced proline content (Table 1). The catalytic loop containing the essential histidine residue was notably longer in Est2, comprising 18 amino acids compared to just 13 in EstC (Figure 8B). Structural measurements showed Est2 possesses a substantially larger catalytic cavity measuring 308.5 Å^3^, more than double the volume of EstC’s 124.9 Å^3^ cavity, and features additional substrate-access tunnels (Figure 8C).

**Table 1:** Parameters affecting thermo-stability and flexibility.

Parameters	Est2	EstC
Amino acids	338	301
No. proline residues/%	22/6.5	26/8.6
No. asparagine residues/%	2/1.5	0/0.0
No. lysine residues/%	9/2.7	2/0.7
No. methionine residues/%	4/1.2	2/0.3
The amount of glycine near the catalytic pocket	5	0
Total accessible surface area (Å^2^)	15461.6	12850.9
Non polar surface area (Å^2^)	9404.5	7634.9
Polar surface area (Å^2^)	2685.9	2145.2
Binding pocket volume (Å^3^)	308.5	124.9

A particularly significant finding was the differential distribution of glycine residues between the two enzymes. While the two enzymes contained a similar number of glycine residues, Est2 uniquely had five glycine residues positioned near its catalytic pocket, a structural feature absent in EstC that likely enhances flexibility at low temperatures. Surface analysis further demonstrated Est2’s greater molecular accessibility, with its total solvent-accessible surface area (15,461.6 Å^2^) being significantly larger than EstC’s (12,850.9 Å^2^). Notably, Est2’s non-polar accessible surface area (9,404.5 Å^2^) also exceeded that of EstC (7,634.9 Å^2^) (Table 1), consistent with its higher content of exposed hydrophobic residues (Figure 8A). These structural modifications, including the strategic glycine positioning, expanded hydrophobic surfaces and optimized cavity architecture, collectively represent specialized evolutionary adaptations that enable Est2 to maintain catalytic efficiency in cold environments.

## 4 Discussion

In this study, we report the identification and biochemical characterization of Est2, a novel cold-adapted esterase. Phylogenetic analysis demonstrated that Est2 forms an independent branch distinct from the 21 known esterase families (Figure 3). According to the ESTHER database, a specialized database for α/β-hydrolase fold proteins; (Lenfant et al., 2013), Est2 was classified into the abh_upf0017 family but not any of the known 21 esterase families. Sequence comparison of Est2 with all 19 members of the bh_upf0017 family revealed an exceptionally low degree of mean sequence identity, only 7.24% (Supplementary Figure S1). Based on these phylogenetic and sequence characteristics, we conclude that Est2 represents a novel esterase family, which we designate as Family XXII.

BLAST analysis identified Est2 as a member of the α/β-hydrolase superfamily. Three-dimensional modeling revealed a classical α/β hydrolase fold architecture, consisting of 9 β-sheets and 10 α-helices (Figure 1). Sequence alignment confirmed the presence of both the conserved GXSXG motif and catalytic triad (Ser^141^-Asp^275^-His^303^), characteristic features of this enzyme family (Johan et al., 2021).

The cold-adapted properties of Est2 are particularly noteworthy. Our experiments demonstrated that Est2 maintains 20% of its maximal activity at 0°C, with optimal activity between roughly 15–30°C (Figure 5B). This is a profile typical of cold-active enzymes. Most of the cold-adapted enzymes showed the optimum activity in the temperature range of 10 to 40°C (Santiago et al., 2016), such as 25°C for AT2 lipase from Mesophilic *Staphylococcus epidermidis* (Kamarudin et al., 2014), 40°C for esterase Est19 from the Antarctic Bacterium *Pseudomonas* sp. E2-15 (Bai et al., 2019) and 30°C for Esterase MHlip from an Antarctic soil metagenomic library (Berlemont et al., 2013).

Most cold-adapted enzymes exhibited poor thermal stability at above 40°C and their activity decreases with increasing temperature. Est2 is no exception, being rapidly inactivated above 40°C (Figure 5D). This thermal instability aligns with the classic trade-off observed in cold-adapted enzymes, where enhanced flexibility at low temperatures compromises thermostability (Siddiqui and Cavicchioli, 2006). Notably, Est2 retained only about 40% activity after 2 h at 20°C, contrasting with thermostable esterases like ThLip1 and ThLip2 from *Thermoanaerobacterium thermosaccharolyticum*, which reported ThLip1 maintained approx. 85% of original activity after 2 h incubation at 75°C and ThLip2 possessing 2 h half-life at 80°C (Li et al., 2018).

The substrate specificity profile of Est2 provides further evidence for its classification as an esterase rather than a lipase. Our data show elevated enzymatic activity toward mid-length *p*NP esters (C6-C8), with a pronounced decrease in catalytic efficiency for longer-chain substrates (C12-C16) (Figure 5A). This preference for mid-chain substrates aligns with structural predictions of Est2’s active site, which features a deep and narrow acyl-binding pocket (Figure 8C). This pattern differs from cold-adapted lipases like the cold-active lipase of *Pseudomonas fragi*, which exhibits broader substrate range (Alquati et al., 2002), suggesting distinct ecological roles for these enzymes in polar environments. Notably, Est2’s substrate profile contrasts with other cold-active esterases, such as EstN7 (Noby et al., 2022), which is strictly limited to short-chain substrates (C2–C4) due to a steric “plug” formed by residues M187 and N211 in its shallow acyl pocket. In contrast, Est2’s deeper pocket accommodates C6–C8 esters but still excludes longer chains, resembling the substrate range of the Serratia sp. esterase EstS (Jiang et al., 2016), though EstS exhibits higher activity at C2–C4. These differences highlight how subtle variations in active site architecture—depth, plasticity, and steric barriers—fine-tune esterase specificity, likely reflecting adaptations to distinct substrate niches in cold environments.

Cold-active enzymes typically are active around pH 7–8 with stability ranging from pH 6–9. To date, only a few lipases reported an optimum activity at pH 9–10 (Ganasen et al., 2016). Similarly, Est2 demonstrates notable alkaline tolerance, showing comparable high activity between pH 7.5–9 (Figure 5C) and measurable activity across pH 6–10 – broader than most cold-active esterases. This extended pH range suggests unique structural adaptations, possibly through optimized surface charge distribution, while maintaining its cold-adapted characteristics.

Experimental data demonstrated that Cu^2+^, Co^2+^, and Zn^2+^ (1 mM) strongly inhibited Est2 activity (>80% loss), while K^+^ and Li^+^ had negligible effects (Figure 7A). This inhibition pattern resembles reports for other cold-adapted esterases, such as esterase EstK from *Pseudomonas mandelii* (Lee et al., 2013) where transition metals similarly caused significant activity loss. Notably, Est2 lost most of its activity at 1 mM Mn^2+^ and was nearly inactivated at 10 mM, consistent with the established view that metal ions can restrict enzyme flexibility. However, there are also cases where metal ions directly or indirectly enhance the cold activity of psychrophilic enzymes. For instance, the Antarctic esterase *M*-Est exhibits higher catalytic efficiency and thermostability upon Mn^2+^ binding (Marchetti et al., 2023). Structural analysis revealed that *M*-Est utilizes a conserved surface-exposed M1 site (E10/D115 cluster) to stabilize localized active-site rearrangements. Similarly, the cold-adapted inorganic pyrophosphatase from *Shewanella* sp. AS-11 features a di-Mn^2+^ active center (Horitani et al., 2020). In the substrate-free state, the bridging water molecule remains distant from the Mn^2+^ ions, resulting in weak exchange coupling and a “loose” active-site structure. Upon substrate analog binding, the water molecule moves closer to the metal center, strengthening the exchange coupling and transitioning the active site into an “optimized” state for catalysis. This suggests that Est2 may lack the Mn^2+^-binding site found in *M*-Est, or that Mn^2+^ induces inhibitory conformational changes in Est2 rather than stabilization. These differences highlight the evolutionary plasticity of cold-adapted enzymes in balancing metal sensitivity and environmental adaptation.

Est2 showed complete inactivation in the presence of 20% formaldehyde or acetonitrile. Higher concentrations (40%) of methanol, ethanol and isopropanol also led to complete loss of activity. Notably, Est2 retained 45% activity in 20% DMSO, a level of tolerance comparable to other reported cold-adapted esterases (De Santi et al., 2014; Guo et al., 2016; Rahman et al., 2016).

Based on these biochemical characteristics as well as the phylogenetic and sequence analyses, we propose that Est2 forms a distinct evolutionary branch, of a previously unclassified esterase family (i.e., family XXII). Est2 appears to function as a cold-active enzyme, exhibiting optimal catalytic activity toward medium chain esters with typical psychrophilic thermal lability. Comparative analysis with the thermophilic Family XX esterase EstC revealed distinct cold-adaptation features in Est2. The structural features of Est2 reveal distinct cold-adaptation strategies. Its extended catalytic loop and glycine clustering near the active site enhance flexibility, while reduced proline content decreases structural rigidity – both common adaptations in cold-active enzymes (Hashim et al., 2018). The significantly larger catalytic cavity with additional tunnels likely compensates for reduced molecular motion at low temperatures (Paredes et al., 2011). Notably, Est2’s increased hydrophobic surface exposure helps maintain stability despite cold-induced weakening of hydrophobic interactions (Siddiqui and Cavicchioli, 2006). This combination of structural flexibility and surface hydrophobicity, along with strategic placement of destabilizing residues, represents an evolutionary balancing act that enables catalysis in cold environments.

## Data Availability

The original contributions presented in the study are publicly available. This data can be found in the UniProt (https://www.uniprot.org/) and NCBI GenBank repositories (https://www.ncbi.nlm.nih.gov/genbank/), accession numbers listed in Supplementary Table S1.

## Footnotes

^1^ https://www.ncbi.nlm.nih.gov/orffinder/

^2^ https://services.healthtech.dtu.dk/services/SignalP-6.0/

^3^ https://blast.ncbi.nlm.nih.gov/Blast.cgi

^4^ https://espript.ibcp.fr/ESPript/ESPript/index.php

^5^ https://itol.embl.de/

^6^ http://pymol.sourceforge.net/

## References

[ref1] Alquati C. De Gioia L. Santarossa G. Alberghina L. Fantucci P. Lotti M. (2002). The cold-active lipase of *Pseudomonas fragi*. Heterologous expression, biochemical characterization and molecular modeling. Eur. J. Biochem. 269, 3321–3328. doi: 10.1046/j.1432-1033.2002.03012.x, PMID: 12084074

[ref2] Arpigny J. L. Jaeger K. E. (1999). Bacterial lipolytic enzymes: classification and properties. Biochem. J. 343, 177–183. 10493927 PMC1220539

[ref3] Ashaolu T. J. Malik T. Soni R. Prieto M. A. Jafari S. M. (2025). Extremophilic microorganisms as a source of emerging enzymes for the food industry: a review. Food Sci. Nutr. 13:e4540. doi: 10.1002/fsn3.4540, PMID: 39803234 PMC11716999

[ref4] Bai L.-S. Zhao C.-X. Xu J.-J. Feng C. Li Y.-Q. Dong Y.-L.. (2019). Identification and biochemical characterization of carboxylesterase 001G associated with insecticide detoxification in Helicoverpa armigera. Pestic. Biochem. Physiol. 157, 69–79. doi: 10.1016/j.pestbp.2019.03.009, PMID: 31153479

[ref5] Berlemont R. Jacquin O. Delsaute M. La Salla M. Georis J. Verté F.. (2013). Novel cold-adapted esterase MHlip from an Antarctic soil metagenome. Biology 2, 177–188. doi: 10.3390/biology2010177, PMID: 24832657 PMC4009859

[ref6] Bornscheuer U. T. (2002). Microbial carboxyl esterases: classification, properties and application in biocatalysis. FEMS Microbiol. Rev. 26, 73–81. doi: 10.1111/j.1574-6976.2002.tb00599.x, PMID: 12007643

[ref7] Chiuri R. Maiorano G. Rizzello A. Del Mercato L. L. Cingolani R. Rinaldi R.. (2009). Exploring local flexibility/rigidity in psychrophilic and mesophilic carbonic anhydrases. Biophys. J. 96, 1586–1596. doi: 10.1016/j.bpj.2008.11.017, PMID: 19217874 PMC2717254

[ref8] De Maayer P. Anderson D. Cary C. Cowan D. A. (2014). Some like it cold: understanding the survival strategies of psychrophiles. EMBO Rep. 15, 508–517. doi: 10.1002/embr.201338170, PMID: 24671034 PMC4210084

[ref9] De Santi C. Tedesco P. Ambrosino L. Altermark B. Willassen N.-P. De Pascale D. (2014). A new Alkaliphilic cold-active esterase from the psychrophilic marine bacterium Rhodococcus sp.: functional and structural studies and biotechnological potential. Appl. Biochem. Biotechnol. 172, 3054–3068. doi: 10.1007/s12010-013-0713-1, PMID: 24488777

[ref10] Ganasen M. Yaacob N. Rahman R. N. Z. R. A. Leow A. T. C. Basri M. Salleh A. B.. (2016). Cold-adapted organic solvent tolerant alkalophilic family I.3 lipase from an Antarctic Pseudomonas. Int. J. Biol. Macromol. 92, 1266–1276. doi: 10.1016/j.ijbiomac.2016.06.095, PMID: 27506122

[ref11] Gasteiger E. Hoogland C. Gattiker A. Duvaud S. E. Wilkins M. R. Appel R. D.. (2005). “Protein identification and analysis tools on the ExPASy server” in The proteomics protocols handbook. ed. Walker J. M. (Totowa, NJ: Humana Press), 571–607.

[ref12] Gomes J. Steiner W. (2004). The biocatalytic potential of extremophiles and extremozymes. Food Technol. Biotechnol. 42, 223–235. Available online at: https://ftb.com.hr/archives/554-the-biocatalytic-potential-of-extremophiles-and-extremozymes

[ref13] Guo H. Zhang Y. Shao Y. Chen W. Chen F. Li M. (2016). Cloning, expression and characterization of a novel cold-active and organic solvent-tolerant esterase from Monascus ruber M7. Extremophiles 20, 451–459. doi: 10.1007/s00792-016-0835-9, PMID: 27209523

[ref14] Hashim N. H. F. Mahadi N. M. Illias R. M. Feroz S. R. Abu Bakar F. D. Murad A. M. A. (2018). Biochemical and structural characterization of a novel cold-active esterase-like protein from the psychrophilic yeast Glaciozyma Antarctica. Extremophiles 22, 607–616. doi: 10.1007/s00792-018-1021-z, PMID: 29556723

[ref15] Horitani M. Kusubayashi K. Oshima K. Yato A. Sugimoto H. Watanabe K. (2020). X-ray crystallography and Electron paramagnetic resonance spectroscopy reveal active site rearrangement of cold-adapted inorganic pyrophosphatase. Sci. Rep. 10:4368. doi: 10.1038/s41598-020-61217-6, PMID: 32152422 PMC7062746

[ref16] Javed S. Azeem F. Hussain S. Rasul I. Siddique M. H. Riaz M.. (2018). Bacterial lipases: a review on purification and characterization. Prog. Biophys. Mol. Biol. 132, 23–34. doi: 10.1016/j.pbiomolbio.2017.07.014, PMID: 28774751

[ref17] Jeanmougin F. Thompson J. D. Gouy M. Higgins D. G. Gibson T. J. (1998). Multiple sequence alignment with Clustal X. Trends Biochem. Sci. 23, 403–405. doi: 10.1016/s0968-0004(98)01285-7, PMID: 9810230

[ref18] Jiang H. Zhang S. Gao H. Hu N. (2016). Characterization of a cold-active esterase from Serratia sp. and improvement of thermostability by directed evolution. BMC Biotechnol. 16:7. doi: 10.1186/s12896-016-0235-3, PMID: 26800680 PMC4722774

[ref19] Johan U. U. M. Rahman R. Kamarudin N. H. A. Ali M. S. M. (2021). An integrated overview of bacterial carboxylesterase: structure, function and biocatalytic applications. Colloids Surf. B. Biointerfaces 205:111882. doi: 10.1016/j.colsurfb.2021.111882, PMID: 34087776

[ref20] Jumper J. Evans R. Pritzel A. Green T. Figurnov M. Ronneberger O.. (2021). Highly accurate protein structure prediction with AlphaFold. Nature 596, 583–589. doi: 10.1038/s41586-021-03819-2, PMID: 34265844 PMC8371605

[ref21] Kamaruddin S. Ahmad Redzuan R. Minor N. Seman W. M. Md Tab M. Jaafar N. R.. (2022). Biochemical characterisation and structure determination of a novel cold-active proline iminopeptidase from the psychrophilic yeast, *Glaciozyma antarctica* PI12. Catalysts 12:722. doi: 10.3390/catal12070722

[ref22] Kamarudin N. H. A. Rahman R. N. Z. R. A. Ali M. S. M. Leow T. C. Basri M. Salleh A. B. (2014). A new cold-adapted, organic solvent stable lipase from mesophilic *Staphylococcus epidermidis* AT2. Protein J. 33, 296–307. doi: 10.1007/s10930-014-9560-3, PMID: 24777627

[ref23] Kielkopf C. L. Bauer W. Urbatsch I. L. (2020). Bradford assay for determining protein concentration. Cold Spring Harb. Protoc. 2020:102269. doi: 10.1101/pdb.prot102269, PMID: 32238597

[ref24] Kim J. T. Kang S. G. Woo J. H. Lee J. H. Jeong B. C. Kim S. J. (2007). Screening and its potential application of lipolytic activity from a marine environment: characterization of a novel esterase from Yarrowia lipolytica CL180. Appl. Microbiol. Biotechnol. 74, 820–828. doi: 10.1007/s00253-006-0727-5, PMID: 17119955

[ref25] Kovacic F. Babic N. Krauss U. Jaeger K.-E. (2018). “Classification of lipolytic enzymes from bacteria” in Aerobic utilization of hydrocarbons, oils and lipids. ed. Fernando R. (Cham: Springer International Publishing), 1–35.

[ref26] Lee C. Kim J. Hong S. Goo B. Lee S. Jang S.-H. (2013). Cloning, expression, and characterization of a recombinant esterase from cold-adapted *Pseudomonas mandelii*. Appl. Biochem. Biotechnol. 169, 29–40. doi: 10.1007/s12010-012-9947-6, PMID: 23117417

[ref27] Lenfant N. Hotelier T. Velluet E. Bourne Y. Marchot P. Chatonnet A. (2013). ESTHER, the database of the α/β-hydrolase fold superfamily of proteins: tools to explore diversity of functions. Nucleic Acids Res. 41, D423–D429. doi: 10.1093/nar/gks1154, PMID: 23193256 PMC3531081

[ref28] Letunic I. Bork P. (2024). Interactive tree of life (iTOL) v6: recent updates to the phylogenetic tree display and annotation tool. Nucleic Acids Res. 52, W78–w82. doi: 10.1093/nar/gkae268, PMID: 38613393 PMC11223838

[ref29] Li W. Shi H. Ding H. Wang L. Zhang Y. Li X.. (2018). Characterization of two novel thermostable esterases from *Thermoanaerobacterium thermosaccharolyticum*. Protein Expr. Purif. 152, 64–70. doi: 10.1016/j.pep.2018.04.010, PMID: 29684442

[ref30] Li P. Y. Zhang Y. Q. Zhang Y. Jiang W. X. Wang Y. J. Zhang Y. S.. (2020). Study on a novel cold-active and halotolerant monoacylglycerol lipase widespread in marine Bacteria reveals a new Group of Bacterial Monoacylglycerol Lipases Containing Unusual C(a/S)HSMG catalytic motifs. Front. Microbiol. 11:9. doi: 10.3389/fmicb.2020.00009, PMID: 32038595 PMC6989442

[ref31] Li Q. Q. Zhu Z. R. Liu Q. G. An Y. T. Wang Y. X. Zhang S. B.. (2022). Characterization of a novel thermostable alkaline lipase derived from a compost metagenomic library and its potential application in the detergent industry. Front. Microbiol. 13:1088581. doi: 10.3389/fmicb.2022.1088581, PMID: 36620038 PMC9817002

[ref32] Liu J. Liu W. Xing S. Zhang X. He H. Chen J.. (2021). Diversity of protease-producing bacteria in the soils of the South Shetland Islands, Antarctica. Antonie Van Leeuwenhoek 114, 457–464. doi: 10.1007/s10482-021-01533-7, PMID: 33598877

[ref33] Liu X. Zhou M. Sun R. Xing S. Wu T. He H.. (2022). Characterization of a novel esterase Est33 from an Antarctic bacterium: a representative of a new esterase family. Front. Microbiol. 13:855658. doi: 10.3389/fmicb.2022.855658, PMID: 35655995 PMC9152352

[ref34] Liu X. Zhou M. Xing S. Wu T. He H. Bielicki J. K.. (2021). Identification and biochemical characterization of a novel hormone-sensitive lipase family esterase Est19 from the Antarctic bacterium *Pseudomonas* sp. E2-15. Biomolecules 11:1552. doi: 10.3390/biom11111552, PMID: 34827549 PMC8615396

[ref35] Marchetti A. Orlando M. Mangiagalli M. Lotti M. (2023). A cold-active esterase enhances mesophilic properties through Mn(2+) binding. FEBS J. 290, 2394–2411. doi: 10.1111/febs.16661, PMID: 36266734

[ref36] Nandanwar S. K. Borkar S. B. Lee J. H. Kim H. J. (2020). Taking advantage of promiscuity of cold-active enzymes. Appl. Sci. 10:8128. doi: 10.3390/app10228128

[ref37] Noby N. Johnson R. L. Tyzack J. D. Embaby A. M. Saeed H. Hussein A.. (2022). Structure-guided engineering of a family IV cold-adapted esterase expands its substrate range. Int. J. Mol. Sci. 23:4703. doi: 10.3390/ijms23094703, PMID: 35563094 PMC9100969

[ref38] Panda T. Gowrishankar B. S. (2005). Production and applications of esterases. Appl. Microbiol. Biotechnol. 67, 160–169. doi: 10.1007/s00253-004-1840-y, PMID: 15630579

[ref39] Paredes D. I. Watters K. Pitman D. J. Bystroff C. Dordick J. S. (2011). Comparative void-volume analysis of psychrophilic and mesophilic enzymes: structural bioinformatics of psychrophilic enzymes reveals sources of core flexibility. BMC Struct. Biol. 11:42. doi: 10.1186/1472-6807-11-42, PMID: 22013889 PMC3224250

[ref40] Pettersen E. F. Goddard T. D. Huang C. C. Meng E. C. Couch G. S. Croll T. I.. (2021). UCSF ChimeraX: structure visualization for researchers, educators, and developers. Protein Sci. 30, 70–82. doi: 10.1002/pro.3943, PMID: 32881101 PMC7737788

[ref41] Rafeeq H. Hussain A. Shabbir S. Ali S. Bilal M. Sher F.. (2022). Esterases as emerging biocatalysts: mechanistic insights, genomic and metagenomic, immobilization, and biotechnological applications. Biotechnol. Appl. Biochem. 69, 2176–2194. doi: 10.1002/bab.2277, PMID: 34699092

[ref42] Rahman M. A. Culsum U. Tang W. Zhang S. W. Wu G. Liu Z. (2016). Characterization of a novel cold active and salt tolerant esterase from *Zunongwangia profunda*. Enzym. Microb. Technol. 85, 1–11. doi: 10.1016/j.enzmictec.2015.12.013, PMID: 26920474

[ref43] Santiago M. Ramírez-Sarmiento C. A. Zamora R. A. Parra L. P. (2016). Discovery, molecular mechanisms, and industrial applications of cold-active enzymes. Front. Microbiol. 7:1408. doi: 10.3389/fmicb.2016.01408, PMID: 27667987 PMC5016527

[ref44] Siddiqui K. S. Cavicchioli R. (2006). Cold-adapted enzymes. Annu. Rev. Biochem. 75, 403–433. doi: 10.1146/annurev.biochem.75.103004.142723, PMID: 16756497

[ref45] Teufel F. Almagro Armenteros J. J. Johansen A. R. Gíslason M. H. Pihl S. I. Tsirigos K. D.. (2022). SignalP 6.0 predicts all five types of signal peptides using protein language models. Nat. Biotechnol. 40, 1023–1025. doi: 10.1038/s41587-021-01156-3, PMID: 34980915 PMC9287161

[ref46] Tribelli P. M. López N. I. (2018). Reporting key features in cold-adapted bacteria. Life 8:10008. doi: 10.3390/life8010008, PMID: 29534000 PMC5871940

[ref47] Trifinopoulos J. Nguyen L. T. Von Haeseler A. Minh B. Q. (2016). W-IQ-TREE: a fast online phylogenetic tool for maximum likelihood analysis. Nucleic Acids Res. 44, W232–W235. doi: 10.1093/nar/gkw256, PMID: 27084950 PMC4987875

[ref48] Wang B. Wu S. Chang X. Chen J. Ma J. Wang P.. (2020). Characterization of a novel hyper-thermostable and chlorpyrifos-hydrolyzing carboxylesterase EstC: a representative of the new esterase family XIX. Pestic. Biochem. Physiol. 170:4704. doi: 10.1016/j.pestbp.2020.104704, PMID: 32980065

[ref49] Willard L. Ranjan A. Zhang H. Monzavi H. Boyko R. F. Sykes B. D.. (2003). VADAR: a web server for quantitative evaluation of protein structure quality. Nucleic Acids Res. 31, 3316–3319. doi: 10.1093/nar/gkg565, PMID: 12824316 PMC168972

[ref50] Ye B. Tian W. Wang B. Liang J. (2024). CASTpFold: computed atlas of surface topography of the universe of protein folds. Nucleic Acids Res. 52, W194–w199. doi: 10.1093/nar/gkae415, PMID: 38783102 PMC11223844

