# Supplementary material for: Expression and characterization of Est2: a novel cold-adapted esterase from Antarctic bacterium defining the new esterase family XXII
Source: Front Microbiol. 2025 Sep 5;16:1662394. doi: 10.3389/fmicb.2025.1662394 (PMC12446244; doi:10.3389/fmicb.2025.1662394)
Supplement: Supplementary file 1 [file Supplementary_file_1.docx]

Supplementary Material

## Supplementary Figures


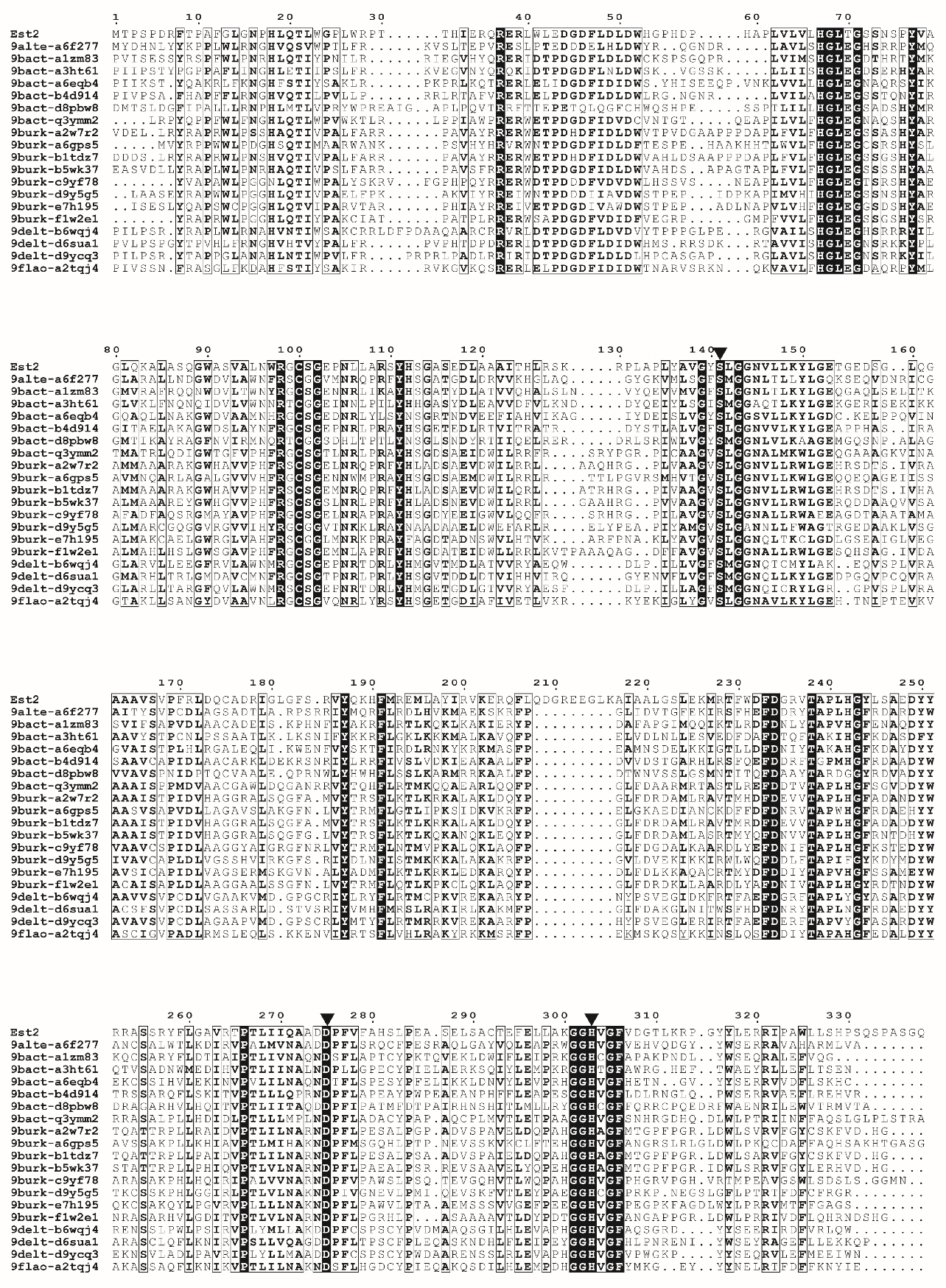


**Figure S1.** **Sequence alignment of Est2 and proteins from abh_upf0017 family.** Catalytic triad residues (Ser-Asp-His) are marked by black triangles. The ESPript 3.0 sequence alignment results showed that the mean sequence identity was 7.24% and the mean similarity was 60.74％.

## Supplementary Table

**Table S1.** Esterases sequences used in the phylogenetic analysis.

| **Organisms** | **UniProt or Genbank Accession No.** | **Family** |
| --- | --- | --- |
| *Proteus vulgaris* | Q52614 | Ⅰ |
| *Pseudomonas fluorescens* | O68310 | Ⅰ |
| *Pseudomonas aeruginosa* | P95419 | Ⅰ |
| *Salmonella typhimurium* | AAC38796.1 | Ⅱ |
| *Photorhabdus luminescens* | CAA47020.1 | Ⅱ |
| *Streptomyces albus* | Q59798 | Ⅲ |
| *Streptomyces* sp. | Q56008 | Ⅲ |
| *Moraxella* sp. (strain TA144) | P19833 | Ⅲ |
| *Dehalococcoidia* bacterium | A0A2E5LY74 | Ⅳ |
| *Pseudomonas* sp. B11-1 | O52270 | Ⅳ |
| *Moraxella* sp. (strain TA144) | P24484 | Ⅳ |
| *Psychrobacter immobilis* | Q02104 | Ⅴ |
| *Moraxella* sp. (strain TA144*)* | P24640 | Ⅴ |
| *Arthrospira platensis* | Q53415 | Ⅵ |
| *Pseudomonas fluorescens* | Q53547 | Ⅵ |
| *Bacillus subtilis* (strain 168) | P37967 | Ⅶ |
| *Streptomyces coelicolor* (strain ATCC BAA-471) | Q9RKZ7 | Ⅶ |
| *Pseudarthrobacter oxydans* | Q01470 | Ⅶ |
| *Streptomyces anulatus* | O87861 | Ⅷ |
| *Pseudomonas fluorescens* | Q53403 | Ⅷ |
| *Arthrobacter globiformis* | Q44050 | Ⅷ |
| *Lysobacter* sp. Root494 | A0A0Q7Q3S1 | Ⅸ |
| *Paucimonas lemoignei* | Q939Q9 | Ⅸ |
| *Shewanella halifaxensis* (strain HAW-EB4) | B0TLS4 | Ⅸ |
| *Thermotoga* sp. RQ7 | A0A0B5KXQ5 | Ⅹ |
| *Thermotoga maritima* (strain ATCC 43589 ) | Q9WYH1 | Ⅹ |
| *Trichodesmium erythraeum* (strain IMS101) | UPI00003C9FC4 | Ⅺ |
| *Rhodopirellula baltica* (strain SH1) | UPI00001ACC08 | Ⅺ |
| *Colwellia psychrerythraea* | UPI000056E257 | Ⅺ |
| *Marinomonas* sp. MED121 | UPI0000690BA8 | Ⅻ |
| uncultured bacterium | B1PZ93 | Ⅻ |
| *Clostridium acetobutylicum* | UPI00000CA890 | Ⅻ |
| *Geobacillus stearothermophilus* | Q06174 | XIII |
| *Alkalihalobacillus pseudofirmus* (strain OF4) | D3FY90 | XIII |
| *Thermoanaerobacterium* | UPI0001B0C2CD | XIV |
| *Caldanaerobacter subterraneus* | UPI00000D6E2D | XIV |
| *Bacillus sp.* (strain H-257) | P82597 | XV |
| *Geobacillus thermodenitrificans* | G3JWZ2 | XV |
| uncultured bacterium | K7QE06 | XV |
| *Xanthomonas euvesicatoria* | UPI00005CE776 | XVI |
| *Saccharothrix espanaensis* | UPI00028BB641 | XVI |
| *Stenotrophomonas maltophilia* | S4TNY8 | XVI |
| *Arsenicicoccus* sp. oral taxon 190 | UPI00067D7D19 | XVII |
| *Dermatophilus congolensis* | UPI0004203A07 | XVII |
| *Janibacter* sp. R02 | A0A1S5R222 | XVII |
| *Solibacillus silvestris* | UPI0002045A9D | XVIII |
| *Lysinibacillus manganicus* | UPI000531A963 | XVIII |
| *Stenotrophomonas maltophilia* | UPI000710186B | XIX |
| *Stenotrophomonas maltophilia* | A0A218L045 | XIX |
| *Stenotrophomonas pavanii* | UPI00088576A2 | XIX |
| *Streptomyces* sp. NRRL WC-3753 | KPC70032.1 | XX |
| *Streptomyces viridosporus* ATCC 14672 | EFE68124.1 | XX |
| *Streptomyces rochei* | BBC94648.1 | XX |
| *Pseudomonas* sp. E5-12 | Est33 MZ717198 | XXI |
| *Pseudomonas arsenicoxydans* | WP 090184031 | XXI |
| *Pseudomonas fluorescens* group | psefs-c3k3m8 | XXII |
| *Pseudomonas protegens* CHA0 | psef5-q4k4b4 | XXII |
| *Pseudomonas* sp*.* | psepf-q3k576 | XXII |
| ***Pseudomonas* sp. A6-5** | **Est2 OR552631** | **XXII** |
